# Prostaglandin I_2_ Signaling Drives Th17 Differentiation and Exacerbates Experimental Autoimmune Encephalomyelitis

**DOI:** 10.1371/journal.pone.0033518

**Published:** 2012-05-10

**Authors:** Weisong Zhou, Dustin R. Dowell, Matthew M. Huckabee, Dawn C. Newcomb, Madison G. Boswell, Kasia Goleniewska, Matthew T. Lotz, Shinji Toki, Huiyong Yin, Songyi Yao, Chandramohan Natarajan, Pingsheng Wu, Subramaniam Sriram, Richard M. Breyer, Garret A. FitzGerald, R. Stokes Peebles

**Affiliations:** 1 Division of Allergy, Pulmonary and Critical Care Medicine, Department of Medicine, Vanderbilt University School of Medicine, Nashville, Tennessee, United States of America; 2 Division of Neurology, Department of Medicine, Vanderbilt University School of Medicine, Nashville, Tennessee, United States of America; 3 Division of Clinical Pharmacology, Department of Medicine, Vanderbilt University School of Medicine, Nashville, Tennessee, United States of America; 4 Division of Nephrology, Department of Medicine, Vanderbilt University School of Medicine, Nashville, Tennessee, United States of America; 5 Department of Medicine and Pharmacology, University of Pennsylvania, Philadelphia, Pennsylvania, United States of America; Universität Würzburg, Germany

## Abstract

**Background:**

Prostaglandin I_2_ (PGI_2_), a lipid mediator currently used in treatment of human disease, is a critical regulator of adaptive immune responses. Although PGI_2_ signaling suppressed Th1 and Th2 immune responses, the role of PGI_2_ in Th17 differentiation is not known.

**Methodology/Principal Findings:**

In mouse CD4^+^CD62L^+^ naïve T cell culture, the PGI_2_ analogs iloprost and cicaprost increased IL-17A and IL-22 protein production and Th17 differentiation *in vitro*. This effect was augmented by IL-23 and was dependent on PGI_2_ receptor IP signaling. In mouse bone marrow-derived CD11c^+^ dendritic cells (BMDCs), PGI_2_ analogs increased the ratio of IL-23/IL-12, which is correlated with increased ability of BMDCs to stimulate naïve T cells for IL-17A production. Moreover, IP knockout mice had delayed onset of a Th17-associated neurological disease, experimental autoimmune encephalomyelitis (EAE), and reduced infiltration of IL-17A-expressing mononuclear cells in the spinal cords compared to wild type mice. These results suggest that PGI_2_ promotes *in vivo* Th17 responses.

**Conclusion:**

The preferential stimulation of Th17 differentiation by IP signaling may have important clinical implications as PGI_2_ and its analogs are commonly used to treat human pulmonary hypertension.

## Introduction

Prostaglandin I_2_ (PGI_2_) is a lipid product of arachidonic acid metabolism and signals through a seven transmembrane G_s_ protein-coupled receptor known as IP [1]. PGI_2_ is produced in greatest abundance by vascular tissues and signaling through IP protects against vascular remodeling and thrombogenesis [2]. PGI_2_ and its analogs with longer biologic half-lives are currently being used therapeutically in patients with primary pulmonary hypertension, as well as in other causes of pulmonary hypertension, including scleroderma, systemic lupus erythematosus, congenital heart disease, HIV, and Gaucher’s disease [3]. In addition to its vascular effects, PGI_2_ is also an important mediator of inflammation. Signaling through IP inhibited Th1 inflammation in a mouse model of respiratory syncytial virus infection and blunted Th2 inflammation in murine allergen challenge models [4]–[8]. However, the role of PGI_2_ in modulating Th17 inflammation has not been completely described.

Th17 cells are distinct from Th1 and Th2 cells and are associated with autoimmune diseases, such as multiple sclerosis and rheumatoid arthritis [9]. Cytokines responsible for the differentiation of naïve mouse T cells into Th17 cells are IL-6 and TGF-β [10]–[12]. IL-23 produced by dendritic cells also plays a pivotal role in the development of Th17 cells. *In vitro* studies revealed that IL-23 promoted the survival of Th17 cells, maintained IL-17A production and induced IL-22 expression [13], [14]. Another study in mice further indicated that IL-23 was required for driving terminal Th17 differentiation [15]. IL-23 was essential for *in vivo* expansion of pathogenic Th17 cells in mouse models of autoimmune inflammation as indicated by undetectable IL-17-producing T cells in IL-23 p19 deficient mice [13], [16]. In experimental autoimmune encephalomyelitis (EAE), an animal model of human multiple sclerosis, IL-23 and Th17 cells were critical for the induction, but not the effector phase, of EAE [16]. In addition, compared to wild type (WT) mice, IL-17A knockout (KO) mice had significantly suppressed EAE as indicated by delayed disease onset, reduced maximum severity scores, attenuated histological changes, and early recovery from the disease [17].

Th17 cell differentiation and proliferation is negatively regulated by the Th1 cytokine IFN-γ and the Th2 cytokines IL-4 and IL-13 [18], [19]. Anti-IFN-γ, anti-IL-4 and anti-IL-13 antibodies increased IL-17A production by CD4 T cells polarized with TGF-β and IL-6 [18], [19]. Consistently, the STAT4 and STAT6 signaling pathways critical for Th1 and Th2 differentiation, respectively, inhibit Th17 differentiation [18]. We previously published that the PGI_2_ analogs cicaprost and iloprost inhibited bone marrow derived dendritic cell (BMDC) production of IL-12, a critical factor in Th1 development, as well as blunted the ability of dendritic cells to generate an antigen-specific Th2 response [20]. We further reported that these PGI_2_ analogs inhibited the production of IFN-γ by polarized Th1 cells and suppressed IL-4 and IL-13 expression by polarized Th2 cells in a dose-dependent pattern [21]. Since PGI_2_ inhibited production of cytokines known to negatively regulate Th17 production, we hypothesized that PGI_2_ promotes Th17 development and cytokine production.

## Materials and Methods

### Ethics Statement

All experimental protocols were approved by Institutional Animal Care and Use Committee at Vanderbilt University (Protocol # M/05/316).

### Mice

Female BALB/c, C57BL/6 and OT II mice were obtained from The Jackson Laboratory. IP KO mice were generated by homologous recombination in embryonic stem cells and were backcrossed to a C57BL/6 background for >10 generations [22]. OT II-IP KO mice were generated by breeding IP KO mice with OT II mice. Age-matched C57BL/6 and OT II mice were used as control mice for IP KO and OT II-IP KO mice, respectively. The mice were used at 8–12 weeks old.

### Reagents

Cicaprost was a gift from Dr. M. Huebner (Schering-Plough Corporation). Iloprost was obtained from Cayman Chemicals. Recombinant IL-4, anti-CD3 (clone 2C11) and anti-CD28 (37.51) were from BD Biosciences. IL-23 and GM-CSF was obtained from R&D Systems. Neutralizing anti-IL-4 and anti-IFN-γ antibodies and rat IgG1 were from BD Biosciences.

### Naïve CD4^+^CD62L^+^ T Cell Culture and Treatment

CD4^+^CD62L^+^ cells were obtained from mouse spleens with mouse naïve CD4^+^CD62L^+^ T cell isolation kits (Miltenyi Biotec). These cells were resuspended at 1×10^6^ cells/ml in RPMI-1640 medium (Mediatech, Inc.) supplemented with 10% FBS (HyClone), 4 mM L-glutamine, 1 mM sodium pyruvate, 55 µM 2-mercaptoethanol, 10 mM HEPES, 100 units/ml penicillin and 100 µg/ml streptomycin. CD4^+^CD62L^+^ T cells of OT II and OT II-IP KO mice were stimulated with ovalbumin peptide 323–339 (OVA_323–339_) (1 µg/ml) and anti-CD28 (1 µg/ml) in 96-well plates for 4 days.

CD11c^+^ cell-depleted CD4^+^CD62L^+^ cells of OT II, C57BL/6, and BALB/c mice were purified by Miltenyi CD4^+^CD62L^+^ T cell purification kit with an additional step to remove CD11c^+^ cells with biotin-conjugated anti-CD11c antibody (BD Biosciences) and streptavidin-Microbeads (Miltenyi). CD11c^+^ cell-depleted CD4^+^CD62L^+^ cells were stimulated with plate-bound anti-CD3 and anti-CD28 in 96-well plates for 4 days [21]. To prepare antibody-bound plates, sodium bicarbonate buffer (0.1 M, pH9.6) containing anti-CD3 (5 µg/ml) and anti-CD28 (2 µg/ml) was added to the plate (50 µl/well). The plates were incubated at 37°C for >4 h and washed twice with RPMI 1640 before cells were seeded.

To determine the effect of PGI_2_ on T cell differentiation, we used the PGI_2_ analogs iloprost and cicaprost with longer biologic half-lives than PGI_2_ as PGI_2_ is very unstable in aqueous solution. Iloprost and cicaprost were added at 10-fold serial-diluted concentrations (1 nM, 10 nM and 100 nM) to the culture medium at the beginning of the cell culture. Vehicle solutions (methyl acetate for iloprost and water for cicaprost) were used as control treatments. All concentrations of the same PGI_2_ analog were adjusted to contain same amount of vehicle. In some experiments, IL-23 (10 ng/ml), IL-4 (10 ng/ml), anti-IL-4 (10 µg/ml), and/or anti-IFN-γ (10 µg/ml) were added to the cell culture in addition to PGI_2_ analogs at the time of T cell activation.

### Flow Cytometry

CD4^+^CD62L^+^ cells isolated from OT II mouse spleens with Miltenyi CD4^+^CD62L^+^ T cell purification kit were stained with propidium iodide and either Alexa Fluor-labeled anti-CD11c antibody (eBioscience) or control rat isotype IgG2a-Alexa Fluor. The cells were analyzed by BD™ LSR II flow cytometer (BD Bioscience). In some experiments, CD4^+^CD62L^+^CD11c^−^ cells of C57BL/6 mouse spleens were purified, activated with anti-CD3 and anti-CD28, and treated with iloprost or cicaprost at 100 nM in the presence of IL-23 (10 ng/ml) at the beginning of the cell culture. The cells were cultured for 4 days and treated with GolgiPlug, PMA (1 ng/ml) and ionomycin (1 µM) for 6 h before being harvested. The cells were stained with Live/Dead Cell Viability Assay Kit (Invitrogen), anti-IL-17A and anti-CD4 for flow cytometry.

### Cytokine Measurements by ELISA and ELISPOT

IL-17A, IL-22, IL-4 and IFN-γ were measured by Quantikine and Duoset ELISA kits (R&D Systems) according to the manufacturer’s instructions. The ability of cells to secrete IL-17A was analyzed by ELISPOT kits (MabTech Inc.). Briefly, after OT II and OT II-IP KO CD4^+^CD62L^+^ cells were activated and differentiated with OVA_323–339_ and anti-CD28 in the presence of PGI_2_ analogs and IL-23 (10 ng/ml) for 4 days, the cells were washed twice and seeded at 2.5×10^5^ cells/ml with OVA_323–339_ (1 µg/ml) in ELISPOT plates coated with IL-17A capture antibody. The cells were cultured for 20 h followed by ELISPOT assay according to manufacturer’s recommendations.

### Dendritic Cell Culture and BMDC-T Cell Co-culture

Bone marrow-derived dendritic cells (BMDCs) were generated using a previously described method [20]. Briefly, the bone marrow in femurs and tibias of OT II and OT II-IP KO mice was flushed out with RPMI 1640 medium and a single-cell suspension was prepared by passing the bone marrow solution through a 19-gauge needle five times. After lysis of RBC, the cells were passed through a nylon cell strainer with a mesh size of 70µm. The cells were then washed and resuspended at 5×10^5^ cells/ml in complete RPMI 1640 medium containing 5% FBS, 50 µg/ml gentamicin, and 55 µM 2-mercaptoethanol. GM-CSF was added to the cell solution at 20 ng/ml. The cells were seeded at day 0 in 6-well plates (2 ml/well) and cultured at 37°C in humidified air containing 5% CO_2_. On day 3, 2 ml of complete medium containing 20 ng/ml of GM-CSF was added to each well. On day 6, half of the culture medium was replaced with complete medium containing 20 ng/ml GM-CSF. At day 8, non- and loosely adherent cells were harvested. Greater than 60% of the harvested cells were CD11c^+^. CD11c^+^ cells were further purified from the mixed cell population with Miltenyi anti-CD11c Microbeads. The purified cells (designated BMDCs) were >94% CD11c^+^ as assessed by flow cytometry. BMDCs were treated with LPS (1 µg/ml) and OVA protein (100 µg/ml) in the presence of iloprost, cicaprost or vehicle solutions, and cultured for 20 h. The culture supernatant was harvested for IL-23 and IL-12 cytokine measurements by ELISA. For BMDC-T cell co-culture experiments, BMDCs were treated with LPS, OVA protein and iloprost (100 nM), washed 3 times to remove iloprost and co-cultured with OT II CD4^+^CD62L^+^ T cells purified with Miltenyi CD4^+^CD62L^+^ T cell purification kit at 1∶2.5 ratio (20,000 BMDCs : 50,000 T cells) for 4 days. The co-culture supernatant was harvested for IL-17A analyses by ELISA.

### Experimental Autoimmune Encephalomyelitis (EAE)

IP KO mice and WT C57BL/6 mice were subcutaneously immunized with 20 µg of myelin oligodendrocyte glycoprotein (MOG) peptide 35–55 (Sigma) in 100 µl PBS emulsified with an equal volume of Freund’s complete adjuvant containing 5 mg/ml *Mycobacterium tuberculosis* H37RA (Difco). The mice were injected with 100 ng of pertussis toxin in 100 µl PBS (List Biological Laboratories) intraperitoneally on days 0 and 2 after immunization. The mice were monitored every day and scored for clinical severity for 24 days on the following scale:1, limp tail; 2, limp tail and weakness of hind legs; 3, limp tail and complete paralysis of hind legs (most common) or limp tail with paralysis of one front and one hind leg; 4, limp tail, complete hind leg and partial front leg paralysis; and 5, complete hind and complete front leg paralysis, no movement around the cage, or dead. Spinal cords were harvested on day 13 after MOG immunization. The mice were perfused with 12 ml PBS before spinal cord harvesting. The spinal cords were minced and the spinal cord cells were isolated with Neural Tissue Dissociation Kits (Miltenyi) following manufacturer’s instructions. The spinal cord cells were resuspended in 0.9 M sucrose in HBSS (Mediatech) and pelleted at 850×g for 10 min. The mononuclear cells of the spinal cords in the pellet were washed and counted. The isolated cells (4×10^5^/ml) were stimulated with PMA (1 ng/ml) and ionomycin (1 µM) for 24 h. The culture supernatant was analyzed for IL-17A production by ELISA.

### Measurement of 2,3-dinor-6-keto-PGF_1α_ in Mouse Urine

C57BL/6 mice were immunized with either MOG peptide/CFA or with saline as a control (15 mice per group). The mice were placed in metabolic cages and 3 mice were placed in each cage. Mouse urine was collected daily for measurements of 2,3-dinor-6-keto-PGF1α, a stable metabolite of PGI_2_, by a gas chromatographic-mass spectrometric assay as previously described [23]. Creatinine in the urine was measured by a chemical assay based on Jaffe’s reaction according to the manufacturer’s instructions (Exocell Inc). The levels of 2,3-dinor-6-keto-PGF_1α_ was normalized to creatinine concentrations in the urine samples.

### Statistics Analysis

The *P* values were calculated by using Student’s *t*-test or one-way ANOVA. Values below the limit of detection were assigned a value that was half the lower limit of detection for that assay. Values of *P*<0.05 were considered significant.

## Results

### PGI_2_ Analogs Induce IL-17A Production by Naïve CD4 T Cells of OT II Mice

To test the hypothesis that PGI_2_ has stimulatory effects on Th17 differentiation, we used CD4^+^CD62L^+^ naïve T cells from spleens of OT II mice for antigen-specific T cell activation and differentiation. OT II CD4 T cells express a transgenic T cell receptor that specifically recognizes the ovalbumin peptide 323–339. Since OT II mice were not exposed to OVA protein before being used in this study, T cells with transgenic TCR specific for OVA_323–339_ should therefore be naïve cells. We used OVA_323–339_ to stimulate OT II CD4^+^CD62L^+^ cells to further ensure that only naïve T cells expressing the transgenic TCR were stimulated and activated. Therefore, we could use this system to determine the effect of PGI_2_ analogs on Th17 differentiation of naïve CD4 T cells.

When OT II CD4^+^CD62L^+^ naïve T cells were stimulated with OVA_323–339_ and anti-CD28 antibody, the cells were activated, proliferated and produced cytokines including IL-4 (529±105 pg/ml), IFN-γ (406±89 pg/ml) and IL-17A (58±26 pg/ml) during 4 days of cell culture after stimulation. Stimulation of OT II CD4^+^CD62L^+^ cells with bovine serum albumin (BSA) and anti-CD28 antibody did not result in cell activation or production of detectable levels of IL-4, IFN-γ and IL-17A. These data indicate that the OT II T cell response to OVA_323–339_ was antigen-specific.

The activation of OT II CD4^+^CD62L^+^ T cells by OVA_323–339_ and anti-CD28 suggested the presence of antigen presenting cells in the CD4^+^CD62L^+^ cell population because presentation of OVA peptide in MHC II molecules is required for CD4 T cell activation. Indeed, we found that 3% of the CD4^+^CD62L^+^ cell population were CD11c^+^ (Figure S1). Since we used 200,000 total cells per well in 96 well plates for the T cell culture, there were approximately 6,000 CD11c^+^ cells per well. When the CD11c^+^ cells were depleted from the CD4^+^CD62L^+^ cell population, the CD11c^+^ cell-depleted CD4^+^CD62L^+^ cells were no longer activated by stimulation with OVA_323–339_ and anti-CD28 and did not produce a detectable level of IL-17A (Figure S2). Therefore the CD11c^+^ cells in the cell population purified by the CD4^+^CD62L^+^ isolation kit acted as antigen presenting cells. We will use the term CD11c^+^ cell-containing CD4^+^CD62L^+^ cells to describe the CD4^+^CD62L^+^ cell population isolated by Miltenyi CD4^+^CD62L^+^ T cell purification kit in this report.

In our study, we found consistent and robust T cell activation and proliferation in CD11c^+^ cell-containing OT II CD4^+^CD62L^+^ cell culture after stimulation with OVA_323–339_ and anti-CD28. This provided us a T cell activation system to study the effect of PGI_2_ analogs on Th17 differentiation in an antigen-specific manner. We used this system to test the hypothesis that PGI_2_ analogs increased Th17 differentiation and reasoned that if we found a pro-IL-17A effect of PGI_2_ analogs on CD11c^+^ cell-containing CD4^+^CD62L^+^ cell culture system, we would then further determine whether PGI_2_ analogs act on CD11c^+^ DCs or on naïve T cells, or both for their pro-Th17 function in vitro.

**Figure 1:**
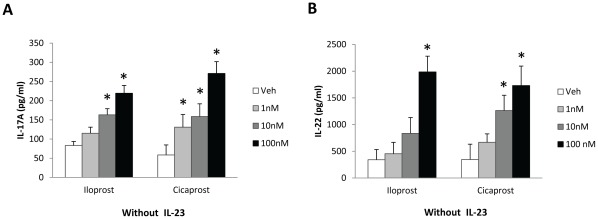
PGI_2_ analogs increased IL-17A and IL-22 production by CD4 T cells. CD11c^+^ cell-containing CD4^+^CD62L^+^ cells isolated from spleens of OT II mice were activated with OVA_323–339_ (1 µg/ml) and anti-CD28 (1 µg/ml) and treated with iloprost, cicaprost, or the respective vehicles as controls for 4 days. The levels of (A) IL-17A and (B) IL-22 in the culture supernatant were determined by ELISA. * p<0.05 vs. vehicle, n = 4. Data (mean ± SEM) are representative of 4 experiments.

To test the hypothesis that PGI_2_ promoted Th17 differentiation, we stimulated CD11c^+^ cell-containing CD4^+^CD62L^+^ cells with OVA_323–339_ and anti-CD28 and treated the cells with the PGI_2_ analogs iloprost, cicaprost or the respective vehicles as controls at the beginning of the cell culture. Four days after T cell activation, the cell culture supernatant was collected for IL-17A measurements by ELISA. As shown in Figure 1A, iloprost and cicaprost dose-dependently increased IL-17A production by the T cells. Treatment with iloprost (100 nM) resulted in a 2.6-fold increase of IL-17A production compared to vehicle treatment (219±50 pg/ml vs. 83±25 pg/ml, p<0.05). Similarly, cicaprost treatment (100 nM) increased IL-17A production 4.6-fold compared to vehicle treatment (270±75 pg/ml vs. 59±64 pg/ml, p<0.05) (Figure 1A). Moreover, these two PGI_2_ analogs also dose-dependently increased the production of IL-22 (Figure 1B), another signature cytokine produced by Th17 cells. Cells treated with iloprost (100 nM) produced 5.8-fold more IL-22 than vehicle-treated cells (1985±195 pg/ml vs.341±191 pg/ml, p<0.05), while cells treated with cicaprost (100 nM) induced 5.0-fold more IL-22 than vehicle-treated cells (1730±365 pg/ml vs. 347±286 pg/ml, p<0.05) (Figure 1B). The stimulatory effect of iloprost and cicaprost on IL-17A and IL-22 production by the *in vitro* stimulated naïve CD4 T cells indicates that the PGI_2_ analogs promoted Th17 differentiation.

### IL-23 Increases the pro-Th17 Effect of PGI_2_ Analogs

**Figure 2:**
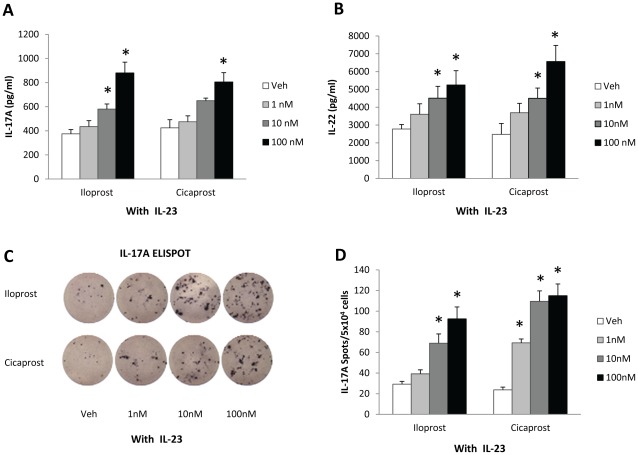
PGI_2_ analogs increased IL-17A and IL-22 production and Th17 differentiation in the presence of IL-23. CD11c^+^ cell-containing CD4^+^CD62L^+^ cells isolated from spleens of OT II mice were activated with OVA_323–339_ (1 µg/ml) and anti-CD28 (1 µg/ml) in the presence of IL-23 (10 ng/ml) and treated with iloprost, cicaprost, or respective vehicles for 4 days. The levels of (A) IL-17A and (B) IL-22 in the culture supernatant were determined by ELISA. (C–D) Iloprost and cicaprost increased the number of IL-17A producing cells and augmented the levels of IL-17A production at a single cell level. At day 4 after activation and differentiation, the cells were washed twice and re-stimulated with OVA_323–339_ (1 mg/ml) for 20 h for IL-17A ELISPOT assay. (C) Representative IL-17A spots. (D) Quantitative presentation of the numbers of spots. * p<0.05 vs. vehicle, n = 4. Data (mean ± SEM) are representative of 4 experiments (A and B) or 3 experiments (C and D).

Since IL-23 promotes Th17 cell survival and expansion [12], [24], we then assessed whether IL-23 further increases IL-17A production with PGI_2_ analogs. We treated CD11c^+^ cell-containing CD4^+^CD62L^+^ cells of OT II mice with OVA_323–339_ and anti-CD28 in the setting of increasing doses of iloprost, cicaprost or the respective vehicles in the presence of IL-23. As shown in Figure 2A, in the presence of IL-23, iloprost and cicaprost further increased IL-17A production in a dose-dependent fashion, compared to vehicles. Iloprost (100 nM) induced 2.3-fold more IL-17A than vehicle (880±89 pg/ml vs. 375±35 pg/ml, p<0.05), while cicaprost (100 nM) induced 1.9-fold more IL-17A than vehicle (805±78 pg/ml vs. 425±67 pg/ml, p<0.05). Similarly, iloprost and cicaprost further augmented IL-22 production up to 2-fold in a dose-dependent manner (Figure 2B). These results indicate that both PGI_2_ analogs and IL-23 stimulate IL-17A and IL-22 expression and that the effects of the individual PGI_2_ analog and IL-23 on IL-17A production appeared to be additive (Figure 1 and Figure 2).

Avni and colleagues previously reported that when naïve CD4 T cells were activated, the cells produced Th1 and Th2 cytokines in a lineage-non-specific manner during the first two days upon TCR-stimulation and costimulatory signaling [25]. It is possible that IL-17A in PGI_2_ analog-treated cell culture was produced transiently before Th17 differentiation. To assess the effect of PGI_2_ analogs on Th17 differentiation, we used ELISPOT to determine the IL-17A-producing ability of CD4^+^CD62L^+^ cells. CD11c^+^ cell-containing CD4^+^CD62L^+^ cells were activated and differentiated with OVA_323–339_, anti-CD28 and IL-23 in the presence of iloprost, cicaprost or the respective vehicles for 4 days. The cells were washed twice and stimulated with OVA_323–339_ for 20 h followed by ELISPOT assay. As seen in Figure 2C and 2D, iloprost and cicaprost dose-dependently increased the numbers of IL-17A-producing cells as indicated by increased numbers of spots compared to vehicle-treated cells. The treatment of cells with the PGI_2_ analogs also resulted in elevated levels of IL-17A expression at a single cell level as indicated by larger sizes of the spots, compared to vehicle controls. Iloprost (100 nM) induced 3.2-fold more IL-17A-producing cells than the vehicle control (93±12 spots vs. 29±3 spots, p<0.05), while cicaprost (100 nM) generated 4.8-fold more IL-17A-producing cells than vehicle (115±11 spots vs. 24±3 spots, p<0.05) (Figure 2D). Therefore, PGI_2_ analogs drove Th17 differentiation of naïve CD4 T cells and induced IL-17A production.

### PGI_2_ Analogs Increase IL-17A Production Through IP Receptor Signaling

**Figure 3:**
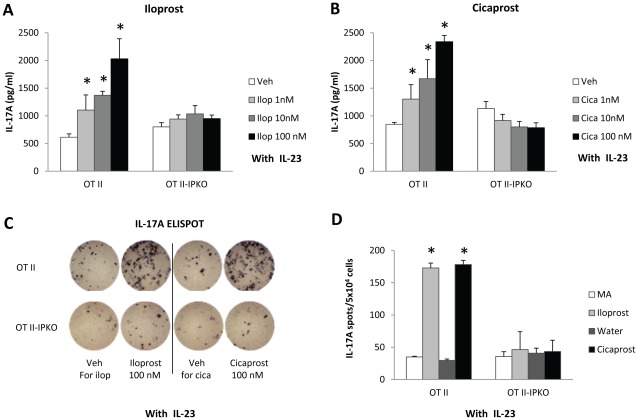
The stimulatory effect of PGI_2_ analogs on IL-17A production and Th17 differentiation was dependent on IP receptor signaling. CD11c^+^ cell-containing CD4^+^CD62L^+^ cells isolated from spleens of OT II mice and OT II-IP KO mice were activated with OVA_323–339_ (1 µg/ml) and anti-CD28 (1 µg/ml) in the presence of IL-23 (10 ng/ml) and treated with iloprost, cicaprost, or respective vehicles for 4 days. (A–B) The levels of IL-17A in the culture supernatant were determined by ELISA. (C and D) The numbers of IL-17A spots in ELISPOT. At day 4 after activation and differentiation, the cells were washed twice and re-stimulated with OVA_323–339_ (1 µg/ml) for 20 h for IL-17A ELISPOT assay. * p<0.05 vs. vehicle, n = 3. Data (mean ± SEM) are representative of 3 experiments.

PGI_2_ signals through IP to increase intracellular cAMP levels and regulate downstream gene expression [20], [26]. To test the hypothesis that the Th17 stimulatory effect of PGI_2_ analogs was mediated by IP receptor signaling in an antigen-specific fashion, we created OT II-IP KO mice that not only express OVA_323–339_-specific TCR but also are deficient in IP receptor. We prepared CD11c^+^ cell-containing CD4^+^CD62L^+^ cells from OT II and OT II-IP KO mouse spleens, activated the cells with OVA_323–339_ and anti-CD28 in the presence of IL-23 and treated the cells with iloprost, cicaprost, or the respective vehicles. As expected the PGI_2_ analogs significantly increased IL-17A production by OT II T cells in a dose-dependent fashion up to 3.4-fold compared to the respective vehicles (Figure 3A and 3B). In contrast, iloprost and cicaprost did not increase IL-17A production by OT II-IP KO T cells (Figure 3A and 3B), indicating that the pro-IL-17A effect of the PGI_2_ analogs was dependent on IP receptor signaling. Moreover, as determined by ELISPOT, the PGI_2_ analogs increased the number of IL-17A-producing cells in OT II T cells, but not in OT II-IP KO cells, after the cells were activated for 4 days and re-stimulated with OVA_323–339_ for 20 h (Figure 3C and 3D), indicating that PGI_2_ analog-induced Th17 differentiation was dependent on IP receptor signaling.

### Recombinant IL-4 Suppressed the Stimulatory Effect of PGI_2_ Analogs on IL-17A Expression

**Figure 4:**
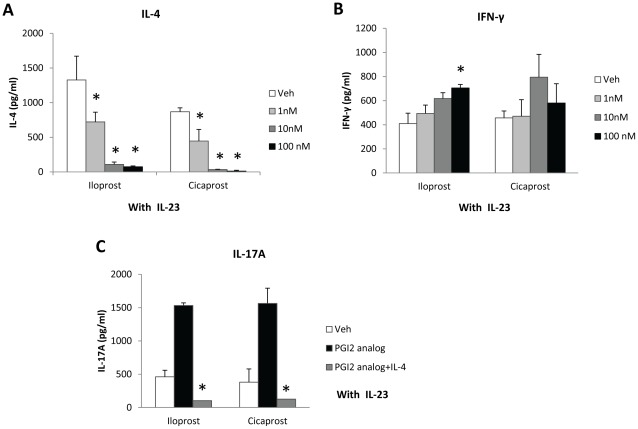
Recombinant IL-4 suppressed the stimulatory effect of PGI_2_ analogs on IL-17A expression. CD11c^+^ cell-containing CD4^+^CD62L^+^ cells isolated from spleens of OT II mice were activated with OVA_323–339_ (1 µg/ml) and anti-CD28 (1 µg/ml) in the presence of IL-23 (10 ng/ml) and treated with iloprost, cicaprost, or respective vehicles for 4 days. (A and B) Iloprost and cicaprost decreased IL-4 production, but not IFN-γ production as determined by ELISA. (C) Iloprost and cicaprost did not have pro-IL-17A effect in the presence of exogenous IL-4. Recombinant IL-4 (10 ng/ml) was added to the culture at the beginning of the cell culture and the levels of IL-17A in the supernatant at day 4 were determined by ELISA. * p<0.05 vs. vehicle (A and B) or vs. PGI_2_ analog (C), n = 3−4. Data (mean ± SEM) are representative of at least 3 experiments.

To determine whether treatment of T cells with PGI_2_ analogs during naïve T cell activation and differentiation affected the profiles of T cell cytokine production, we measured the levels of IL-4 and IFN-γ in the culture supernatant of CD11c^+^ cell-containing CD4^+^CD62L^+^ OT II T cells activated by OVA_323–339_ and anti-CD28 and treated with the PGI_2_ analogs in the presence of IL-23. As shown in Figure 4A and 4B, iloprost and cicaprost inhibited IL-4 production in a dose-dependent fashion. At 10 nM and higher concentrations, the PGI_2_ analogs almost completely suppressed IL-4 production (Figure 4A). However, iloprost and cicaprost did not decrease IFN-γ production and iloprost at 100 nM increased IFN-γ expression (Figure 4B).

Because IL-4 is a strong inhibitor of Th17 differentiation, we hypothesized that the PGI_2_ analogs increased Th17 differentiation by suppressing IL-4 production and Th2 differentiation. To test this hypothesis, we added recombinant IL-4 to the culture of PGI_2_ analog-treated cells. The exogenous IL-4 abrogated the stimulatory effect of iloprost and cicaprost on IL-17A production (Figure 4C), suggesting that activation of the IL-4/IL-4R signaling pathway effectively directed the T cell differentiation away from Th17 lineage. This result also suggests that inhibition of the IL-4/Th2 differentiation pathway is a mechanism by which PGI_2_ analogs promote Th17 differentiation.

### Iloprost Increases the Ratio of IL-23/IL-12 Produced by BMDCs

**Figure 5:**
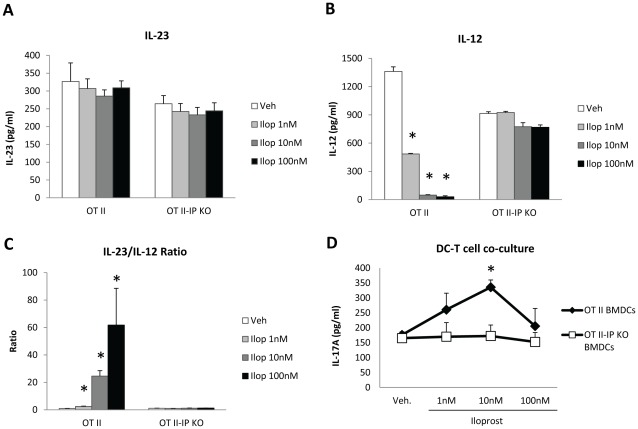
The PGI_2_ analog iloprost increased the ratio of IL-23/IL-12 produced by BMDCs and BMDC’s ability to induce T cell IL-17A responses. Bone marrow-derived DCs of OT II mice and OT II-IP KO mice were generated by culturing bone marrow cells in GM-CSF (20 ng/ml) for 8 days and CD11c^+^ BMDCs were purified by Miltenyi anti-CD11c Microbeads. CD11c^+^ BMDCs were treated with LPS (1 µg/ml), OVA protein (100 µg/ml) and iloprost for 16 h. (A–B) The levels of IL-23 and IL-12 in the culture supernatant were determined by ELISA. (C) The ratio of IL-23/IL-12 in iloprost-treated BMDC culture supernatant. (D) Iloprost-treated OT II BMDCs or OT II-IP KO BMDCs were washed 3 times to remove iloprost and co-cultured with OT II CD4^+^CD62L^+^ cells for 4 days. IL-17A levels in the co-culture supernatant were determined by ELISA. * p<0.05 vs. vehicle (A–C), or vs. OT II-IP KO BMDCs (D). Data (mean ± SEM) are representative of 3 (A–C) and 2 (D) experiments.

The pro-Th17 effect of PGI_2_ analogs on CD11c^+^ cell-containing CD4^+^CD62L^+^ cells suggest that PGI_2_ analogs may promote Th17 differentiation by an indirect effect on DCs or a direct function on T cells, or both. To test the hypothesis that PGI_2_ analogs increased DC’s ability to stimulate T cell differentiation toward Th17 cells, we assessed whether PGI_2_ analogs affected the production of the Th17-driving cytokine IL-23. We generated BMDCs from OT II mice and OT II-IP KO mice by culturing bone marrow cells in GM-CSF for 8 days, followed by purification of CD11c^+^ BMDCs and treatment of the cells with iloprost or vehicle in the presence of LPS and OVA protein. Twenty hours after the treatment, we harvested the culture supernatant for IL-23 and IL-12 assays by ELISA. As shown in Figure 5, iloprost did not change IL-23 production by BMDCs of both OT II and OT II-IP KO mice (Figure 5A). In contrast, iloprost decreased the production of the Th1-driving cytokine IL-12 in OT II BMDCs with almost complete IL-12 suppression at 10 nM and higher concentrations (Figure 5B), compared to the vehicle treatment, consistent with our previously published findings [20]. Iloprost did not change IL-12 production by OT II-IP KO BMDCs (Figure 5B), indicating that the suppressive effect of iloprost on IL-12 was dependent on IP receptor signaling. The differential effects of iloprost on IL-23 and IL-12 production resulted in increased ratio of IL-23/IL-12 in the culture fluid of iloprost-treated OT II BMDCs, compared to that of vehicle-treated OT II BMDCs (Figure 5C).

### Iloprost Increase the Ability of BMDCs to Stimulate T cell IL-17A Production

To further test the hypothesis that PGI_2_ analogs increased the BMDCs’ ability to stimulate T cell IL-17A expression, we treated OT II BMDCs and OT II-IP KO BMDCs with iloprost and OVA protein for 20 h, washed the cells 3 times to remove residual iloprost and OVA protein, and co-cultured the BMDCs with OT II CD4^+^CD62L^+^ cells for 4 days. We found that OT II BMDCs treated with 10 nM iloprost, but not with 100 nM of iloprost, significantly increased IL-17A protein expression in the co-culture supernatant compared to vehicle-treated OT II BMDCs (Figure 5D). Iloprost-treated OT II-IP KO BMDCs did not have augmented IL-17A production in the co-culture experiments compared to vehicle-treated OT II-IP KO BMDCs (Figure 5D), indicating that the stimulatory effect of iloprost on BMDCs’ Th17-induction potential was IP-dependent. Culture of iloprost-treated BMDCs alone did not result in detectable IL-17A production (data not shown), suggesting that IL-17A in the BMDC-T cell co-culture supernatant was produced by CD4^+^CD62L^+^ T cells.

### PGI_2_ Analogs Increased IL-17A Production by A Direct Action on Naïve T Cells

**Figure 6:**
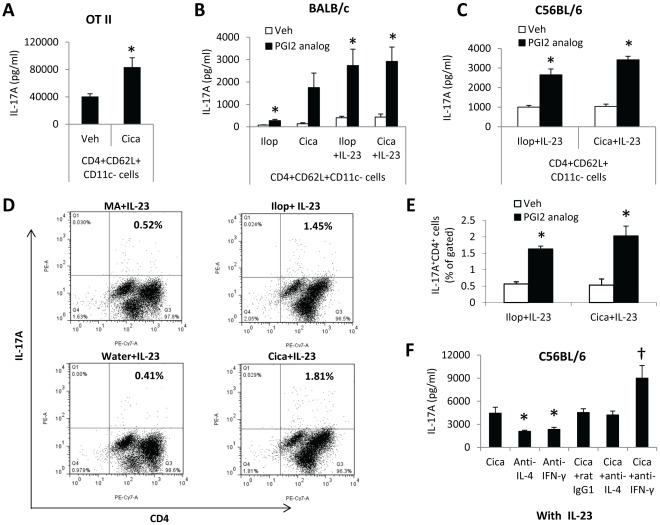
PGI_2_ analogs increased IL-17A production by CD11c^+^ cell-depleted CD4^+^CD62L^+^ cells. CD11c^+^ cell-depleted CD4^+^CD62L^+^ cells isolated from spleens of OT II mice (A), BALB/c mice (B), and C56BL/6 mice (C–F) were activated with plate-bound anti-CD3 (5 µg/ml) and anti-CD28 (2 µg/ml) in the absence or presence of IL-23 (10 ng/ml) for 4 days. The cells were treated with iloprost (100 nM) and cicaprost (100 nM) or the respective vehicles at the beginning of the cell culture. IL-17A production in the culture supernatant was determined by ELISA (A–C and F). Intracellular IL-17A expression was analyzed by flow cytometry and gated for live cells (D and E). * p<0.05 vs. vehicle, n = 4−5 (A–C and F); or n = 3 (E). * p<0.05 vs. cicaprost and † p<0.05 vs. cicaprost plus rat IgG1 control (F). Data (mean ± SEM) are representative of 1 (A), 3 (B), and 2 (C–F) experiments.

After we found that iloprost increased BMDC’s ability to stimulate IL-17A responses of T cells, we investigated whether PGI_2_ analogs had direct effects on T cell IL-17A production. We used CD11c^+^ cell-depleted CD4^+^CD62L^+^ cells of OT II mice and stimulated the cells with anti-CD3 and anti-CD28 antibodies. We used the pan-TCR stimulation because CD11c^+^ cell-depleted CD4^+^CD62L^+^ cells were not activated by OVA_323–339_ and anti-CD28 (Figure S2). As shown in Figure 6, we found that cicaprost increased IL-17A production by CD11c^+^ cell-depleted CD4^+^CD62L^+^ cells of OT II mice (Figure 6A), indicating that cicaprost acted directly on T cells to promote IL-17A production. We also activated CD11c^+^ cell-depleted CD4^+^CD62L^+^ T cells of WT BALB/c or C56BL/6 mice with anti-CD3 and anti-CD28 in the presence or absence of IL-23 and treated the cells with iloprost, cicaprost or the respective vehicles for 4 days. We found that iloprost and cicaprost increased IL-17A production by the cells of BALB/c mice in the absence or presence of IL-23, compared to respective vehicle controls (Figure 6B). Similarly, PGI_2_ analogs increased IL-17A production by CD11c^+^ cell-depleted CD4^+^CD62L^+^ T cells of C56BL/6 mice after stimulation with anti-CD3 and anti-CD28 in the presence of IL-23 (Figure 6C). These results indicate that PGI_2_ analogs augmented IL-17A production by direct action on naïve T cells. The stimulatory effect of PGI_2_ analogs on IL-17A production by pan-TCR-stimulated CD4 T cells in both C57BL/6 (OT II and WT C57BL/6 mice) and BALB/c mouse genetic backgrounds supports that the PGI_2_ analog-driven Th17 differentiation is mouse strain-independent. Furthermore, we determined IL-17A expression of CD11c^+^ cell-depleted CD4^+^CD62L^+^ T cells of WT C56BL/6 mice after the cells were stimulated with anti-CD3 and anti-CD28 in the presence of IL-23 by flow cytometry. We found that PGI_2_ analogs significantly increased the number of IL-17A-expressing cells (Figure 6D and 6E). This finding supports that PGI_2_ analogs promoted Th17 differentiation from naïve CD4 T cells.

To further study whether the pro-Th17A effect of PGI_2_ was mediated by inhibiting the IL-4/Th2 differentiation pathway, we added anti-IL-4 neutralizing antibody to the cell culture in the presence of IL-23. We found that CD11c^+^ cell-depleted CD4^+^CD62L^+^ cells treated with cicaprost plus anti-IL-4 produced similar levels of IL-17A compared to cells treated with cicaprost plus rat IgG1 control (Figure 6F), suggesting that cicaprost and anti-IL-4 did not have an additive pro-IL-17A effect and that the suppression of IL-4 production by cicaprost seems to be a mechanism of the pro-Th17 effect. As a control, neutralization of IFN-γ resulted in a significant increase in IL-17A production by cicaprost-treated cells compared to IgG1 control (Figure 6F), indicating that cicaprost did not augment IL-17A expression by inhibiting IFN-γ.

### IP KO Mice have Delayed EAE Disease Onset

**Figure 7:**
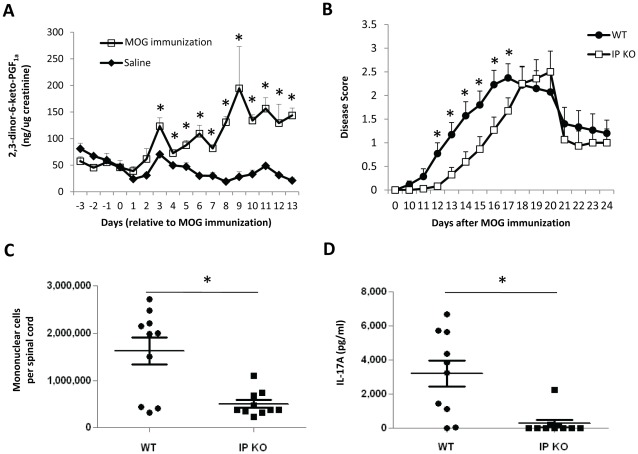
Signaling through IP increased disease development in IL-17A-mediated EAE. IP KO mice and WT C57BL/6 mice were injected with MOG peptide 35–55/CFA and pertussis toxin for EAE induction. (A) PGI_2_ production was elevated during EAE development in WT mice. C57BL/6 mouse urine was collected daily from day −2 to day 13 relative to MOG immunization. The levels of PGI_2_ metabolite, 2,3-dinor-6-keto-PGF_1α_, in the urine was measured by mass spectrometry and normalized to the creatinine levels in the urine as determined by ELISA. (B) IP signaling promoted EAE development. EAE disease scores of WT and IP KO mice were determined based on the neural physical examination. (C and D) IP signaling increased the number and IL-17A responses of mononuclear cells in the spinal cord at day 13 after MOG immunization. Mononuclear cells isolated from the spinal cord were (C) counted and (D) cultured with PMA and ionomycin for 24 h for IL-17A production determined by ELISA. * p<0.05 vs. day 0 (A), or vs. IP KO mice (B, C and D). n = 4 (A, each data point had 3 mice), n = 34–37 mice (B) and 10 mice per group (C and D). Data (mean ± SEM) are representative of 2 experiments (A), 3 experiments (C and D) or combined results of 4 experiments (B).

The *in vitro* pro-Th17 effect of PGI_2_ analogs demonstrated in this study suggests that PGI_2_ plays a role in *in vivo* Th17 differentiation and IL-17A responses. To assess the *in vivo* relevance of the pro-Th17 effect of PGI_2_ analogs, we used WT and IP KO mice in a mouse model of human multiple sclerosis, EAE, a disease associated with IL-17A as IL-17A is important for the early phase of EAE development [27]–[29]. To provide a rationale for this *in vivo* study, we first investigated whether PGI_2_ production was elevated during EAE development by measuring the stable PGI_2_ metabolite, 2,3-dinor-6-keto-PGF_1α_, in mouse urine. As shown in Figure 7A, WT C57BL/6 mice immunized with MOG peptide 35–55 in Freund’s complete adjuvant had significantly elevated levels of PGI_2_ metabolite at multiple time points (day 3 to day 13) compared to saline-injected mice. This result indicates that PGI_2_ synthesis is modulated by EAE, suggesting a role of PGI_2_ in EAE pathogenesis. To examine whether PGI_2_ and IP receptor signaling are involved in EAE development, we used IP KO mice and WT C57BL/6 control mice for EAE induction. As shown in Figure 7B, IP KO mice had significantly delayed disease onset as indicated by greater disease scores for WT mice than IP KO mice from day 12 to day 17. The disease score peaked at day 17 and day 20 for WT mice and IP KO mice, respectively, with comparable peak scores (Figure 7B), indicating that IP deficiency caused a delay of disease onset, but did not affect the peak disease severity. The mice of both strains started to recover from the disease after the peak without further difference in disease scores between WT and IP KO mice (Figure 7B). We also found that the delayed disease onset in IP KO mice correlated with attenuated inflammatory cell infiltration to the spinal cord and decreased IL-17A production by mononuclear cells in the spinal cord (Figure 7C and 7D). There were 3-fold fewer mononuclear cells infiltrated in the spinal cord tissue in IP KO mice than in WT mice (5×10^5^±0.8×10^5^ cells/spinal cord vs. 16.2×10^5^±2.9×10^5^ cells/spinal cord, p<0.05) at day 13 (Figure 7C). When the isolated mononuclear cells of the spinal cords were *in vitro* stimulated with PMA and ionomycin, IP KO cells had significantly decreased IL-17A production, compared to WT cells (Figure 7D). Therefore, IP deficiency resulted in delayed EAE disease onset and blunted inflammation with inhibited IL-17A responses in the spinal cord. These results in EAE experiments suggest that PGI_2_-IP signaling regulates Th17 cell differentiation and expansion in *in vivo* immune responses.

## Discussion

In this study, we report that PGI_2_ enhanced the development of Th17 immune responses and exacerbated a model of Th17-associated neurologic disease, EAE. We found that the presence of PGI_2_ analogs at the time of naïve CD4^+^ T cell differentiation resulted in direct induction of Th17 cytokine secretion, and that this effect was augmented by IL-23. At the antigen presenting cell level, we found that PGI_2_ analogs increased the ratio of IL-23/IL-12 produced by BMDCs. Importantly, the effects of the PGI_2_ analogs on IL-17A cytokine production by naïve CD4^+^ T cells and on the IL-23/IL-12 balance produced by BMDCs were IP-specific. The importance of these *in vitro* findings was confirmed by an *in vivo* model of a Th17-associated disease, EAE, in which IP KO mice were significantly protected against the onset of the neurologic sequelae characteristic of this condition. In these experiments, the inability to signal through IP led to a significant reduction of inflammatory cell infiltration and IL-17 responses in the spinal cord 13 days after disease induction, at a time when there was a significant difference in disease severity between WT and IP KO mice.

We observed that the augmented IL-17A production induced by PGI_2_ analogs correlated with markedly reduced IL-4, but not IFN-γ, production by CD4 T cells during differentiation. When exogenous IL-4 was added to the cell culture, the stimulatory effect of PGI_2_ analogs on IL-17A production was abrogated, suggesting that PGI_2_ analogs enhanced IL-17A production by suppressing IL-4 secretion. The finding that exogenous IL-4 effectively inhibited IL-17A production in the presence of PGI_2_ analogs also implies that IL-4 receptor signaling was not impaired by PGI_2_ analogs. Therefore, PGI_2_ analogs appear to reduce IL-4 secretion rather than block the IL-4 signaling pathway during T cell activation and differentiation. Neutralizing anti-IL-4 antibody did not further increase IL-17A production in the presence of cicaprost compared to IgG1 control treatment (Figure 6F), also suggesting a role of PGI_2_ analog-mediated IL-4 suppression in their pro-Th17A effect. In contrast, neutralization of IFN-γ further increased IL-17A production by cicaprost-treated cells (Figure 6F), suggesting a possible additive effect of cicaprost and anti-IFN-γ on IL-17A expression and cicaprost did not increase IL-17A production by inhibiting IFN-γ.

The PGI_2_ analogs iloprost and cicaprost did not decrease IFN-γ production during primary T cell activation, which is different from the inhibitory effect of those analogs on IFN-γ production by effector T cells in our previous publication [21]. This difference suggests that the function of PGI_2_ analogs on IFN-γ expression appears to be affected by T cell culture conditions and T cell differentiation status. In this study, PGI_2_ analogs were added at the beginning of CD4^+^CD62L^+^ cell culture, while in our previous study [21] PGI_2_ analogs were used to treat CD4^+^ T cells that had been activated and differentiated with anti-CD3 and anti-CD28 for 5 days under Th1 conditions and the analogs were added to the cell culture at the time of restimulation of the differentiated Th1 cells with anti-CD3. Various effects of PGE_2_ on IFN-γ production under different culture conditions were also reported by Yao and colleagues [30]. In their study, PGE_2_ increased IFN-γ production by CD4^+^CD62L^+^ cells under Th1 differentiation condition (IL-2, IL-12 and anti-IL-4), while such stimulatory effect was lost in the absence of the Th1-driving cytokine IL-12 [30].

Our study revealed that CD4^+^CD62L^+^ cell population isolated with Miltenyi CD4^+^CD62L^+^ T cells isolation kit contained 3% CD11c^+^ cells. The CD11c^+^ cells had antigen-presenting function and were responsible for the activation of OT II CD4^+^CD62L^+^ cells stimulated with OVA_323–339_ and anti-CD28. This is supported by the finding that depletion of CD11c^+^ cells from the CD4^+^CD62L^+^ cell population resulted in non-responsiveness of the CD11c^+^ cell-depleted CD4^+^CD62L^+^ cells to OVA_323–339_ and anti-CD28. PGI_2_ analogs increased IL-17A production by CD11c^+^ cell-depleted CD4^+^CD62L^+^ cells activated by anti-CD3 and anti-CD28, indicating that PGI_2_ analogs had a direct effect on naïve CD4 T cell to promote IL-17A expression. The pro-IL-17A effect in our study is supported by Li and colleagues’ recent publication in that PGI_2_ further increased IL-17A production induced by TGF-β and IL-6 in COX-2^−/−^ CD4 T cell culture, and iloprost increased the number of IL-17A-producing CD4 T cells and IL-17A production in the lung in a mouse model of OVA-induced allergic airway inflammation [31].

Our results indicate that PGI_2_ may function as a pro-Th17 agent not only by a direct effect on T cells, but also by indirect effect on DCs. The PGI_2_ analog-treated BMDCs had an increased ratio of IL-23/IL-12 compared to vehicle-treated BMDCs. The greater ratio of IL-23/IL-12 for iloprost (10 nM)-treated BMDCs was correlated with augmented IL-17A production in BMDC-T cell co-culture supernatant, suggesting that iloprost increased BMDCs’ ability to stimulate Th17 differentiation. However, although iloprost at 100 nM also increased IL-23/IL-12 ratio compared to vehicle control, iloprost (100 nM)-treated BMDCs did not induce increased IL-17A production in BMDC-T cell co-culture compared to vehicle-treated BMDCs. This may be because iloprost at high concentrations further inhibited BMDC activation and expression of MHC II molecules and the co-stimulatory molecule CD86 as we previously reported [20].

Similar to PGI_2_ analogs, PGE_2_ has been shown to promote Th17 differentiation of human and mouse CD4 T cells [30], [32]. In human cells, TCR stimulation of CD4 T cells in the presence of PGE_2_ increased IL-17A production [33]. PGE_2_ increased human Th17 cell expansion in the presence of IL-23 [34]. Mouse models of Th17-associated diseases revealed that PGE_2_ regulated IL-17A responses. For instance, *in vivo* administration of PGE_2_ induced IL-23-dependent IL-17A production and administration of the PGE_2_ analog misoprostol exacerbated collagen-induced arthritis [35], [36]. PGE_2_ increased the numbers of CD4^+^IL-17A^+^ T cells and neutrophils in the colonic tissue in a mouse model of experimental inflammatory bowel disease [37]. In LPS-stimulated mouse BMDC culture, PGE_2_ resulted in enhanced IL-23 production and diminished IL-12 secretion [38]. The potent effects of PGI_2_ and PGE_2_ on both DCs and T cells suggest an important role of the products in the arachidonic acid metabolic pathway in immune responses. The differential stimulation of Th17 differentiation by PGI_2_ and PGE_2_ suggests a strong ability of these lipid molecules in regulating immune responses.

The *in vivo* relevance of the *in vitro* effect of PGI_2_ analogs on Th17 differentiation is demonstrated in the current study by delayed disease onset of Th17-associated EAE in IP KO mice compared to WT control mice. The incomplete prevention of EAE development in IP KO mice suggests that the disease pathogenesis is partially dependent on IP signaling. While IP deficiency significantly delayed disease onset, it is not known why the inability to signal through IP did not change the peak disease severity. IP signaling may promote initial generation of IL-17A-producing cells and therefore accelerate the development of EAE, while the magnitude of EAE disease course is not determined by PGI_2_/IP signaling. Consistently, IL-17A-producing Th17 cells were reported to be crucial for the induction of EAE [28], [29] and IL-17A expression correlated with the induction phase and the onset of EAE, but not with the peak disease and resolution phases of EAE [27]. PGI_2_ is abundantly formed by endothelial cells [39] that are present in the lymph tissues. As the levels of PGI_2_ were elevated during EAE development and PGI_2_ signaling accelerated disease onset of EAE, PGI_2_ seems to be actively involved in the development of Th17 responses and the disease pathogenesis. Similarly, signaling through the EP4 receptor of PGE_2_ also contributed to the disease development and IL-17A responses in EAE [30], suggesting a common function of these lipid products in EAE pathogenesis.

The finding that PGI_2_ regulates Th17 differentiation and IL-17A production presents possible important health related issues. PGI_2_ and its analogs are used therapeutically to treat primary pulmonary hypertension, in addition to secondary causes of pulmonary hypertension such as scleroderma, systemic lupus erythematosus, congenital heart disease, HIV, and Gaucher’s disease [3]. Therefore, it is possible that PGI_2_ used to treat pulmonary hypertensive disorders could exacerbate autoimmune conditions presumed to be driven by Th17-associated inflammation such as multiple sclerosis or Crohn’s disease [40]. Whether this occurs clinically is unknown, as to our knowledge, a formal review of the effect of PGI_2_ on these conditions has not been made. On the other hand, IP antagonists might be beneficial in preventing these autoimmune disease states. Further, upregulation of IL-17A production by PGI_2_ might protect against extracellular pathogens, such as *Klebsiella pneumoniae* or *Mycoplasma pulmonis* that require IL-17A to resolve the infection [41], [42]. Specific investigation exploring the *in vivo* role of PGI_2_ in regulating these infections will have to be performed to determine if this eicosanoid modulates immune responses against these organisms *in vivo*.

In conclusion, our study highlights the role of the inflammatory microenvironment as a crucial factor in the regulation of Th17 development. These results add critical information to previous studies which revealed that PGI_2_ negatively regulated Th1 and Th2 helper T cell function and cytokine production, while our investigations revealed that this prostanoid promoted Th17 differentiation highlighting the complexity and fine balance of CD4 differentiation and development.

## Supporting Information

Figure S1 **Presence of CD11c^+^ cells in the CD4^+^CD62L^+^ cell population purified with Miltenyi CD4^+^CD62L^+^ T cell isolation kit.** CD4^+^CD62L^+^ T cells of OT II mice isolated with Miltenyi CD4^+^CD62L^+^ T cell isolation kit were stained with propidium iodide and either Alexa Fluor 647-labeled rat IgG2a or Alexa Fluor 647-labeled anti-CD11c antibody. The cells were analyzed by flow cytometry and gated for PI^–^ live cells.
(TIF)

Figure S2 **CD11c^+^ cell-depleted CD4^+^CD62L^+^cells were activated by pan-TCR stimulation, but not by OVA_323–339_ and anti-CD28.** CD11c^+^ cell-depleted CD4^+^CD62L^+^ cells isolated by Miltenyi CD4^+^CD62L^+^ T cell isolation kit with an additional step to remove CD11c^+^ cells. The cells were cultured with OVA_323–339_ plus anti-CD28 or anti-CD3 plus anti-CD28. (A) Cell images were taken at day 4 after stimulation. Activated and proliferated cells formed colonies after stimulation with anti-CD3 and anti-CD28, but not with OVA_323–339_ and anti-CD28. (B) The level of IL-17A in the culture supernatant collected at day 4 was determined by ELISA. * p<0.05 vs. OVA_323–339_ plus anti-CD28, n = 4.
(TIF)
