# Supplementary figures and images for: Prostaglandin I2 Signaling Drives Th17 Differentiation and Exacerbates Experimental Autoimmune Encephalomyelitis
Source: PLoS One. 2012 May 10;7(5):e33518. doi: 10.1371/journal.pone.0033518 (PMC3349674; doi:10.1371/journal.pone.0033518)

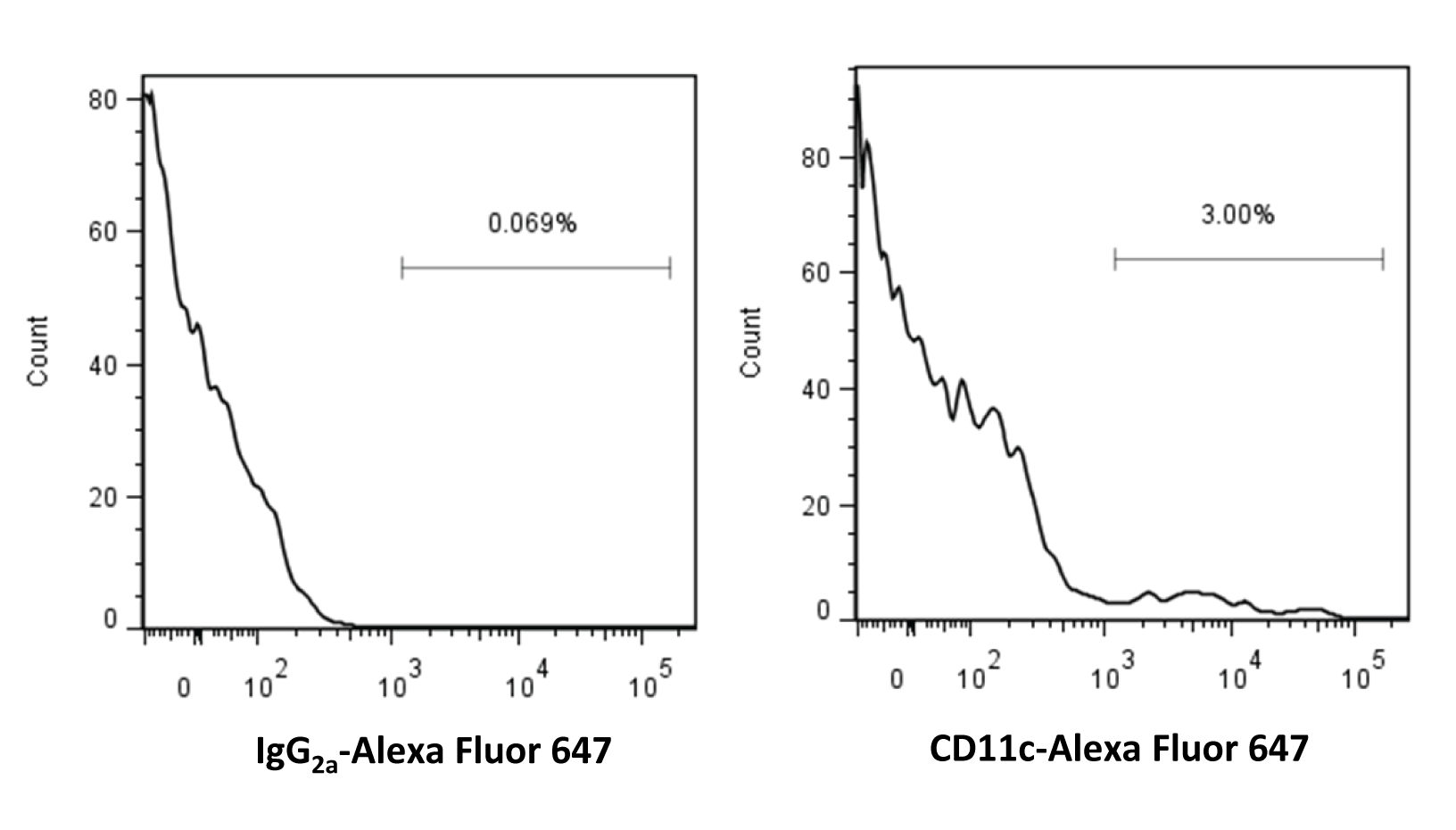

Supplement: Figure S1 — Presence of CD11c+ cells in the CD4+CD62L+ cell population purified with Miltenyi CD4+CD62L+ T cell isolation kit. CD4+CD62L+ T cells of OT II mice isolated with Miltenyi CD4+CD62L+ T cell isolation kit were stained with propidium iodide and either Alexa Fluor 647-labeled rat IgG2a or Alexa Fluor 647-labeled anti-CD11c antibody. The cells were analyzed by flow cytometry and gated for PI– live cells. (TIF) [file pone.0033518.s001.tif]

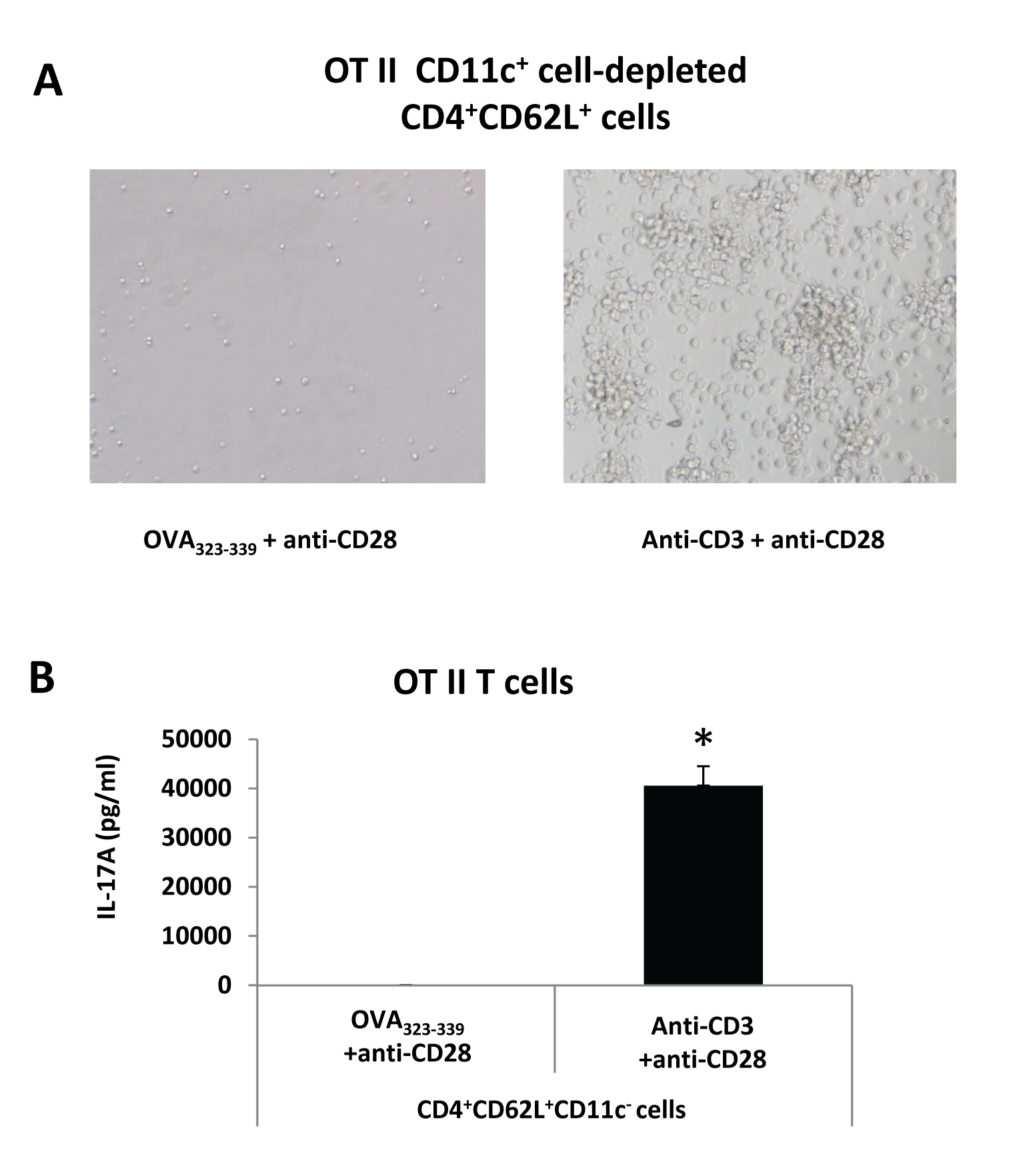

Supplement: Figure S2 — CD11c+ cell-depleted CD4+CD62L+cells were activated by pan-TCR stimulation, but not by OVA323–339 and anti-CD28. CD11c+ cell-depleted CD4+CD62L+ cells isolated by Miltenyi CD4+CD62L+ T cell isolation kit with an additional step to remove CD11c+ cells. The cells were cultured with OVA323–339 plus anti-CD28 or anti-CD3 plus anti-CD28. (A) Cell images were taken at day 4 after stimulation. Activated and proliferated cells formed colonies after stimulation with anti-CD3 and anti-CD28, but not with OVA323–339 and anti-CD28. (B) The level of IL-17A in the culture supernatant collected at day 4 was determined by ELISA. * p<0.05 vs. OVA323–339 plus anti-CD28, n = 4. (TIF) [file pone.0033518.s002.tif]
